# Causal association between employment transitions and suicide in Australia

**DOI:** 10.1038/s41598-025-14217-3

**Published:** 2025-08-17

**Authors:** Swikar Poudel, Sandro Sperandei, Andrew Page, Yi Guo

**Affiliations:** 1 Translational Health Research Institute, Western Sydney University, Campbelltown, 2560 Australia; 2 School of Computer, Data and Mathematical Sciences, Western Sydney University, Parramatta South, 2150 Australia

**Keywords:** Epidemiology, Psychiatric disorders

## Abstract

Suicide remains a significant public health concern globally, with unemployment, financial hardship, and employment transitions cited as contributing factors. However, their causal association remains unclear. This study investigates the causal association between employment transitions and suicide in Australia from 2009 to 2018, utilising Convergent Cross Mapping (CCM), a dynamical systems approach. Employment transitions were categorised into positive transition, no transition, and negative transition groups, representing changes in employment status. Initial analysis of raw data suggested all three groups showed causal association to suicide. However, detrending the time series revealed that only negative transition maintained a significant causal association with suicide, while no transition and positive transition showed no causal association. These findings emphasise the critical association of employment instability on mental health and the importance of stable or improving employment conditions in suicide prevention. The study discusses the need for targeted interventions to mitigate the adverse effects of employment downturns.

## Introduction

Suicide is a serious public health problem with more than 700000 deaths annually^1^. It is the fourth leading cause of death among 15-29 years old globally^1^ and is the leading cause of death for the same age group in Australia^2^. Among many factors that contribute towards this burden, economic factors such as unemployment^3,4^ and financial hardship^5,6^ are often associated with suicide. An economic crisis is often followed by an increase in suicide^7^.

However, the relationship between economic hardship and suicide is known to vary across population subgroups. Theoretical work, such as the concept of the option value of life^8^, suggests that younger individuals may be less likely to respond to economic hardship with suicidal behaviour, as they have a greater perceived capacity to wait for conditions to improve. Empirical evidence has shown that working-age adults, and in particular middle-aged men, tend to be disproportionately affected by unemployment and economic downturns in relation to suicide risk^9–12^.

Studies usually analyse this association using a single employment status and its effect on psychological distress and/or suicide^4,13^. Few studies have examined employment transitions and its effect on mental health outcomes^14,15^. However, there has been limited evidence that observed suicide and economic hardship to have a causal association. It is difficult to define causal association because they emerge from controlled experiments. In cases where it is not feasible to conduct these experiments, alternative methods become crucial to define causal association.

A recent study examined this causal association using unemployment and underutilisation for Australian population using Convergent Cross Mapping (CCM)^16^. CCM is a mathematical model that uses time series information from a dynamical system to imply causation (See Methods for details). The results from the study suggested that there was a significant increase in intentional self-harm and psychological distress with increasing unemployment and underutilisation of the Australian population. The study also discussed that financial hardship, poverty and lower socio-economic status result in poor mental health leading to an eventual suicide.

However, unemployment and underutilisation rate of Australian labour force remained largely steady in the years leading to the onset of COVID-19 pandemic^17^. Therefore, the steady unemployment and underutilisation rate may not capture mechanisms relating to transitions in employment status, and by extension, changes in individuals’ employment status and material circumstances that may be factors that have association with suicide behaviour. As such, this study analysed suicides using employment transitions on Australian population cohort to answer the question, “Is there a causal association between employment transitions and suicide?”

## Results

**Fig. 1:**
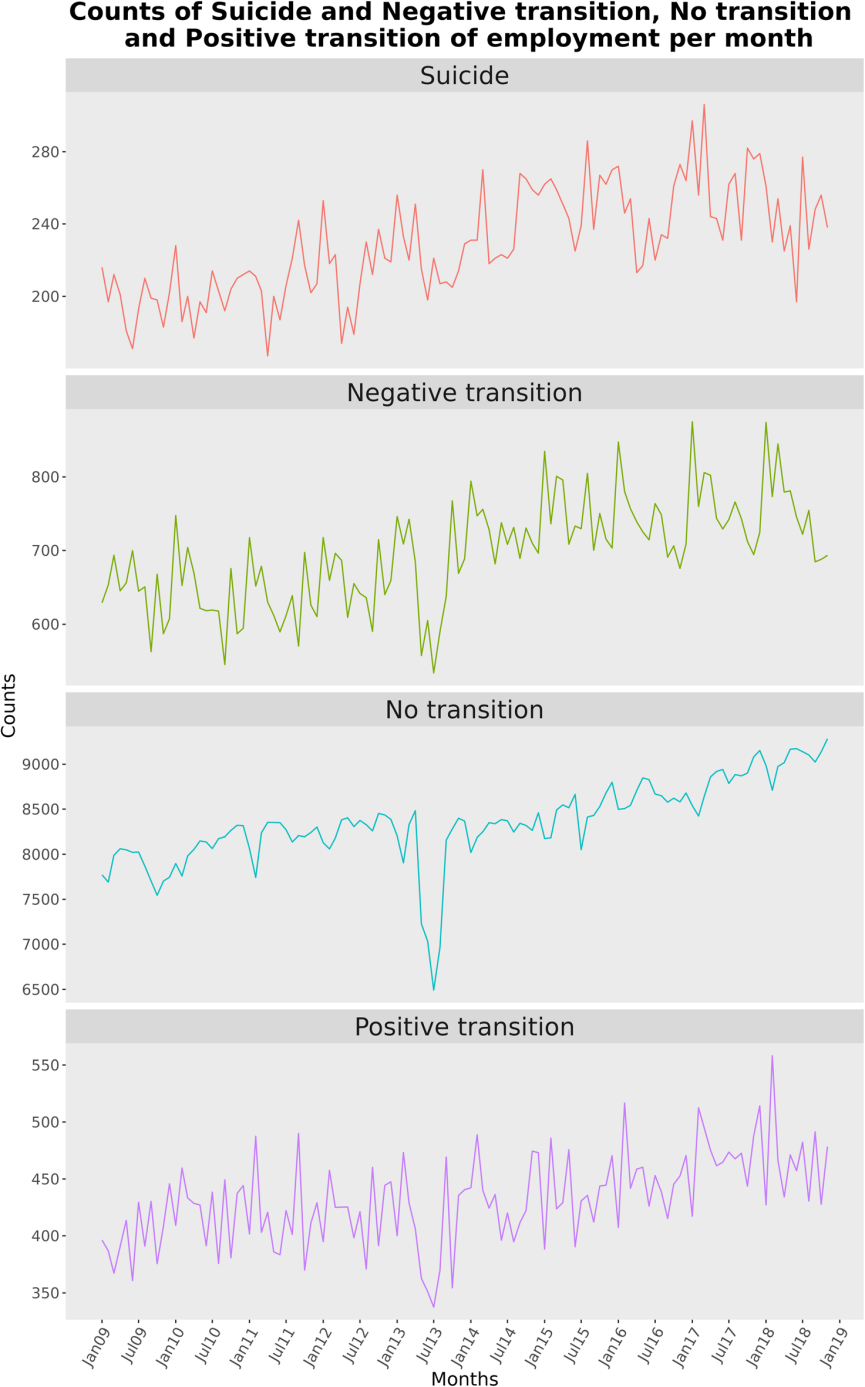
**Monthly count data.** Monthly counts of suicide and population whose economic status had negative transition, no transition and positive transition from January 2009 to November 2018.

**Fig. 2:**
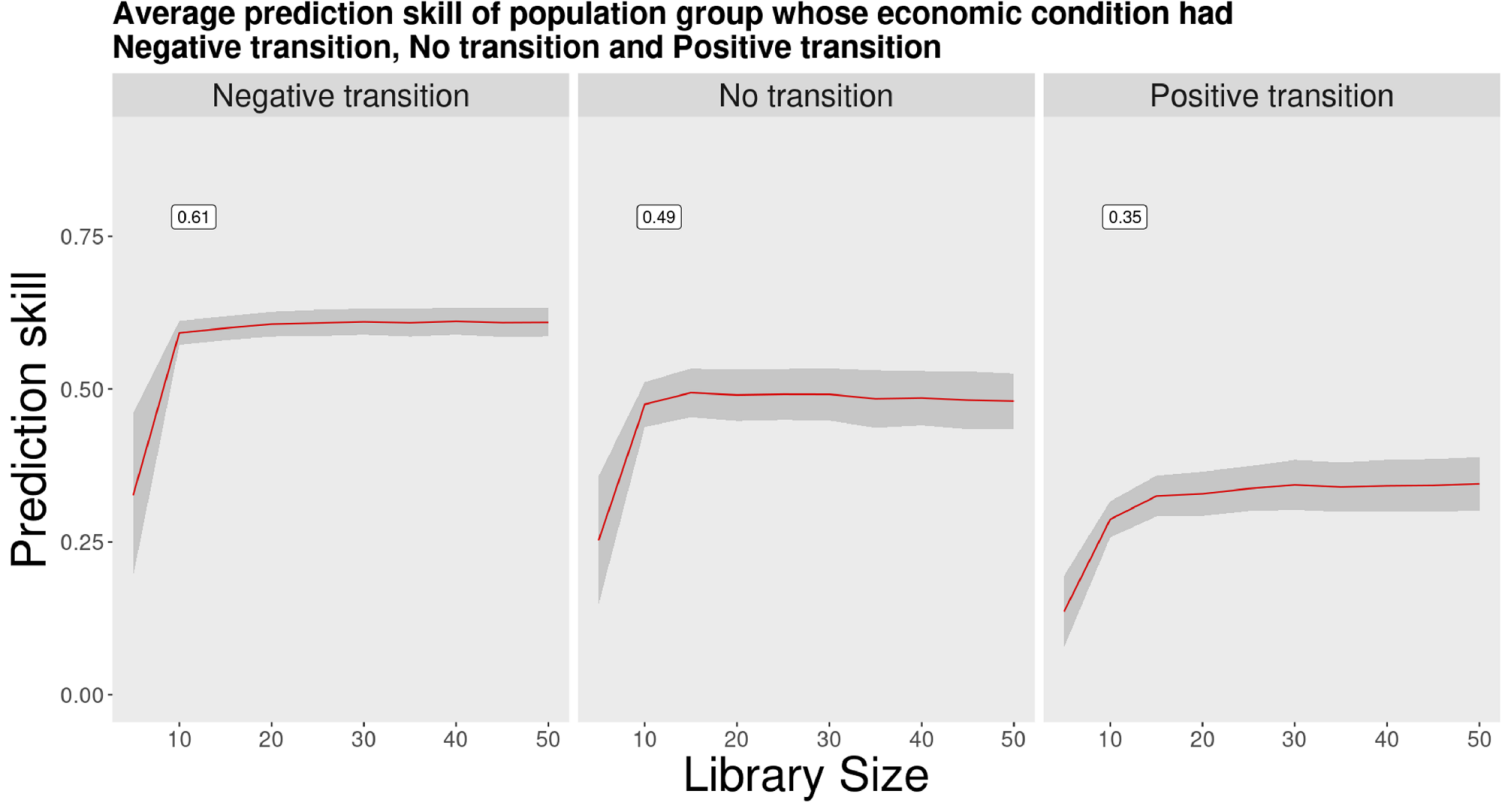
CCM results (raw data). Average prediction skill ($\rho$) of population group whose economic condition had negative transition, no transition and positive transition on their causal association to suicide on raw data. A zero or negative indicates non-causal association and positive indicates a causal association.

Secular trend lines of suicide and employment change were observed in Fig. 1. There was a marked decline in all categories of employment in July 2013 in an otherwise regular monthly fluctuations. The decline in July 2013 was due to the presence of large number of unmatched groups who responded in July 2013 but not in the previous month. The results of CCM analysis suggested that all three employment transition groups– positive transition, no transition, and negative transition–showed varying degrees of causal association on suicide (Fig. 2). The ρ values, which indicate the strength of causality, suggest that all groups were contributing to changes in suicide. A zero or negative ρ﻿ indicates non significant causal association and lacks information across the two time series, whereas positive ρ ﻿indicates a causal association. We found ρ ﻿values of 0.61 for the negative transition group, 0.49 for the no transition group, and 0.35 for the positive transition group. However, given the closed-loop nature of the labour force system, this finding appeared counter-intuitive. In such a system, where one group’s loss should theoretically translate into another’s gain, it is not logical that all groups would simultaneously increase suicide. This prompted the hypothesis that the time series data were not stationary and that the trends within the data might be creating an illusion of causality rather than reflecting true causal associations. To examine whether the observed causal associations were a result of non-stationarity in the time series, we applied a detrending technique to the hypothesis. Prior study in CCM have also endorsed the application of detrending procedure prior to CCM implementation^18^, however, it should be noted that detrending is not an explored concept for CCM analysis. For our analysis, we used a basic linear regression to model the overall trend of the time series and subtracted this trend, effectively to capture the residuals of the time series. Although several other detrending techniques exist, they could potentially overfit the model by estimating more parameters than the simple linear regression approach^19,20^. This approach minimises the risk of losing important signals that are essential for causal detection in CCM. We conducted the Kwiatkowski-Phillips-Schmidt-Shin (KPSS) test to assess the stationarity of the time series before and after the de-trending process. The results indicated that the variables were non-stationary prior to the detrending and stationary post de-trending, supporting the validity of our modelling approach.

**Fig. 3:**
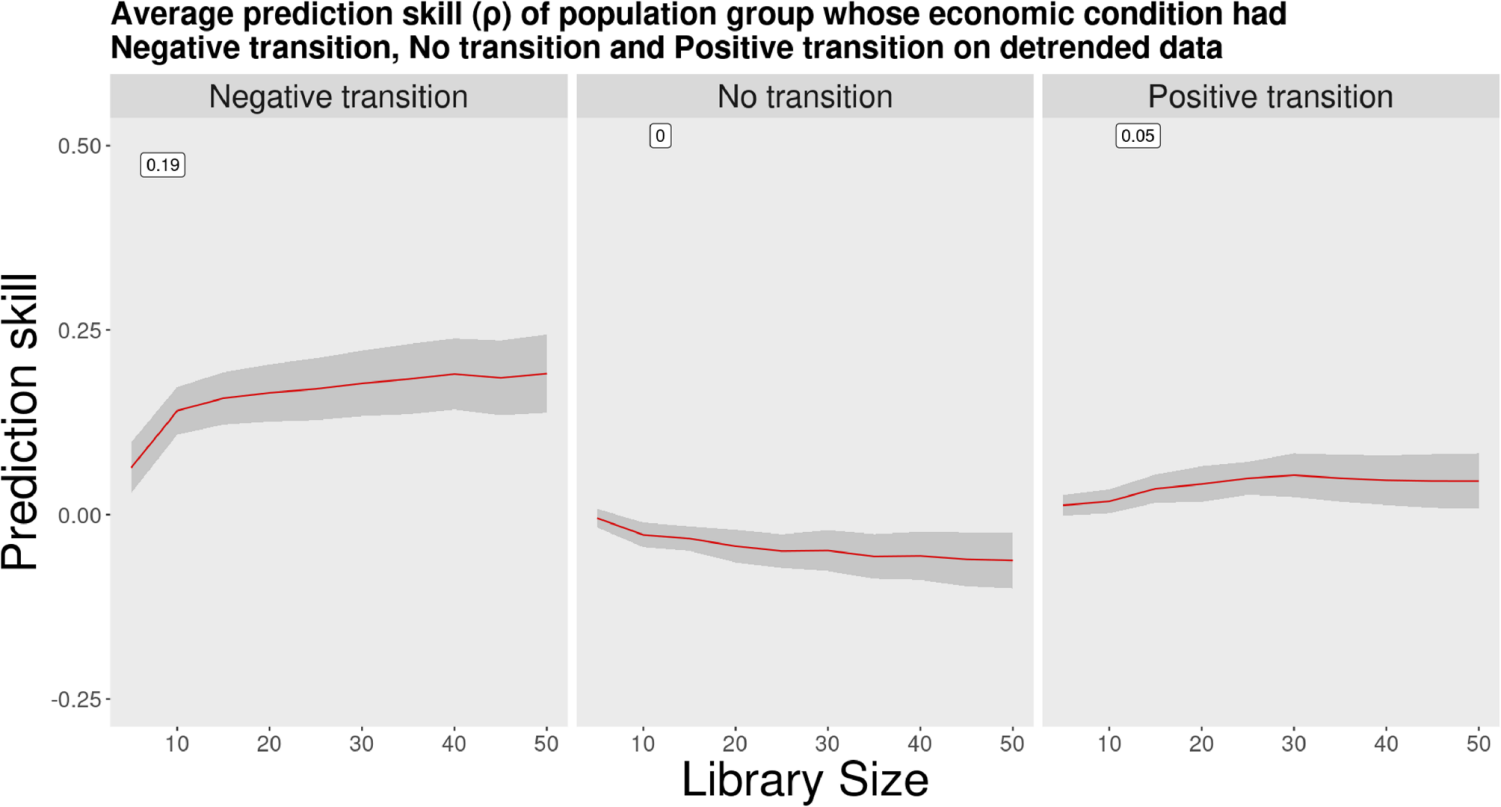
CCM results (detrended data). Average prediction skill ($\rho$) of population group whose economic condition had negative transition, no transition and positive transition on their causal association to suicide on detrended data using linear fit approach. A zero or negative indicates non-causal association and positive indicates a causal association.

After applying CCM to the detrended data, the outcomes indicated a significantly different compared to the raw data. Only the negative transition group (ρ = ﻿0.19) indicates a causal association, while the no transition (ρ = ﻿0) and positive transition (ρ = ﻿0.05) groups indicate no causal association between employment transitions and suicide (Fig. 3). This suggests that a decline in employment status is linked to an increase in suicide, whereas stable or improved employment conditions do not appear to drive suicide. The findings also suggest that the strong causal associations observed in the raw data were likely due to underlying trends rather than actual causality, as evidenced by the significantly lower values in the detrended data.

## Discussion

Our study aimed to investigate if employment transitions have a causal association to suicide. Using Australian cohort population, we have established this link. While negative transitions demonstrate a causal association with increased suicides, no transitions or positive transitions do not exhibit such an association.

Our findings align with^16^ that examined two specific cases of negative transitions–unemployment and underutilization–and found a causal link with suicide. AIHW also estimated lower risk of suicide among those with a job as reported at Census 2011^21^. A US based study in 2020 implemented multiple logistic regression on data from wave 1 (2001 and 2002) and wave 2 (2004 and 2005) of the National Epidemiologic Survey on Alcohol and Related Conditions. The study reported financial debt/crisis, unemployment, and lower income as predictors of suicide attempts^5^. Further, a comprehensive systematic review, encompassing 38 studies conducted between 1992 and 2014, identified that 31 of these studies reported a positive association between macroeconomic factors, such as the unemployment rate and Gross Domestic Product, and suicide rates^22^. Other epidemiological studies also suggest that financial hardship and poverty are related with psychological distress, suicidal ideation and eventual suicide^6,23^.

Employment transitions, and by extension, income and socio-economic position (SEP), have been identified as factors influencing mental health outcomes, which are significant predictors of self-harm and suicide. A study conducted on Australians aged 45 and older from 2006 to 2018 reported that income and employment were mediators of distress or suicide risk^24^, with unemployment and involuntary retirement–both examples of negative transitions–showing a particularly strong direct link to these outcomes^15^. Another study on the same demographic highlighted that negative employment transitions, such as moving from employed to unemployed, retired to unemployed, or remaining unemployed, were strongly associated with psychological distress compared to stable employment^14^. Additionally, a study using data from the Household, Income, and Labour Dynamics in Australia (HILDA) survey reported that individuals in low, lower-middle, or declining SEP groups experienced higher levels of distress and depression or anxiety compared to those in high SEP groups^25^.

Our study suggests that non-negative employment transitions are necessary for suicide prevention, as evident by no causal association between no transition and positive transition with suicide. Individuals having a steady (no transition) or positive employment transition is important and policy makers should contribute towards job security. Studies have suggested financial literacy programme streamlined towards people facing financial hardships could help to better mental health and control suicidal risk^5^. Creating an employment buffer for the underutilised cohort as a means of job guarantee could help people working less hours^16^. Additionally, allowing work from home i.e. being able to work from remote locations helped people from utilising more hours towards work and had better measures of mental health than those who were not employed^26^. For older groups, retirement represents a significant milestone in their life course, and interventions aimed at enhancing their social status and interactions may help mitigate the potential negative effects of employment transitions^15^.

This study investigated the relationship between fluctuations in employment statuses and their association with suicide. However, it is important to note that potential confounding factors such as mental health disorders and prior suicide attempts, which have a multifaceted connection to both suicide and economic hardship^6^, were not accounted for in the analysis. Furthermore, the results of the CCM, as a mathematical model, may be affected by the selection of parameters. In our research, we conducted preliminary investigations to determine the most suitable values in order to mitigate potential bias. Additionally, while our analysis demonstrates causal association, it does not function as a predictive tool; thus, $\rho$ reflects the strength of the association but does not offer predictive outcomes. Consequently, it is not feasible to quantify the number of suicides attributable to each economic condition, which may be essential for policy outcomes. Furthermore, if detrending is crucial for the analysis of the CCM, thorough testing is necessary to validate this assertion. We utilized only linear fit detrending technique; it is possible that employing additional methods could yield different outcomes. Future research may provide further insights into this matter. Despite this, this study answers the question that we sought and we conclude that negative transitions have causal association with suicide while positive transitions and no transitions combat the same.

## Methods

### Suicide and change and employment data

The Australian Institute of Health and Welfare (AIHW) publishes extensive health, disability, and mortality data, including suicide and self-harm monitoring^27^. The monthly suicide data provided by AIHW is based on the International Classification of Diseases (ICD) 10th Revision cause of death codes (X60-84, Y87.0) and covers the period from January 2009 to December 2018.

The Australian Bureau of Statistics (ABS) publishes monthly employment data, which includes both raw and seasonally adjusted values of the change in Australian labour force for individuals aged 15 years and older^17^. Employment status is categorised into six groups: employed full-time, employed part-time, unemployed, not in the labour force, unmatched, and incoming/outgoing rotation group. For this analysis, only the populations categorised as full-time, part-time, and unemployed were considered due to the ambiguity of the other statuses.

With three employment statuses, there are nine possible combinations for changes in employment on a month-to-month basis. These categories were then aggregated into three distinct groups: positive transition, no transition, and negative transition. The positive transition group includes individuals whose employment status improved during the month, such as transitions from unemployed to part-time, unemployed to full-time, and part-time to full-time. The no transition group includes those whose employment status was employed and remained unchanged, such as those who remained in full-time to full-time or part-time to part-time employed. Lastly, the negative transition includes individuals whose employment status declined, such as transitions from full-time to part-time, full-time to unemployed, part-time to unemployed, and unemployed to unemployed. Unemployed to unemployed is categorised as a negative transition instead of no transition due to the lack of securing employment, worsening their financial and economic wellbeing.

### Convergent cross mapping (CCM)

To test the causal association between employment status and suicide, we used convergent cross mapping (CCM). Introduced by Sugihara^28^, CCM has been widely adopted for mapping causal relationship in dynamical systems. CCM uses lagged timestamp co-ordinates of a given nonlinear dynamic system in a higher dimension (*E*) to predict the independent variable using the dependent variable. The higher dimensional reconstruction is referred as shadow manifolds or reconstructed manifolds which contains the historical timestamps of both dependent and independent variable.

Assuming variables *X*(*t*) and *Y*(*t*) are two time series of the same dynamical system and $X(t) \rightarrow Y(t)$, read as *X* causes *Y*, the reconstructed manifolds are created using time lagged co-ordinates of the original time series. If we take $\tau$ as the positive time lag, then the timestamps in the manifold $M_Y(t)$ are constructed as $M_Y(t) = Y(t-\tau ), Y(t-2\tau ), \ldots , Y(t-(E-1)\tau$, similarly for $M_X(t)$.

The historical timestamps of *Y*(*t*) in shadow manifold $M_Y(t)$ i.e. ${Y} = \{M_Y(1), M_Y(2), \ldots , M_Y(L)\}$ can be used to predict the corresponding timestamps of in shadow manifold i.e. , where, is the length of the time segment used for prediction termed as library size.

CCM uses nearest neighbours to provide an approximation for the points in the library of $M_X(t)$ using the corresponding library of $M_Y(t)$. These neighbours are identified using Euclidean distance. The number of nearest neighbours is usually set at default of $E+1$. For each *t*, it predicts the state of *X*(*t*) using the weights based on the distance between $M_Y(t)$ and its neighbours.

$$w_i(t) = \frac{exp(-d(M_Y(t), M_Y(i))/d_{min})}{\sum _{k=1}^{E+1} exp(-d(M_Y(t), M_Y(k))/ d_{min})}$$

where:

*d* is the Euclidean distance between $M_Y(t)$ and $M_Y(i)$

$d_{min}$ is the distance to the nearest neighbour

$w_i(t)$ is the weight assigned to the neighbour *i*

Using these weights, estimate *X*(*t*) as,

$$\hat{X(t)_{M_Y}} = \sum _{i=1}^{E+1} w_i(t)X(i)$$

If $X(t) \rightarrow Y(t)$, the nearest neighbours in $M_Y(t)$ should correspond to nearest neighbours in $M_X(t)$, thus giving a good prediction for *X*(*t*) using $M_Y(t)$. With a predicted value of $\hat{X(t)_{M_Y}}$ and the actual value of *X*(*t*), correlation between the two gives prediction skill, usually represented by $\rho$.

The prediction skill ($\rho$) in CCM is measured by how well the state of one variable can be predicted by another variable over the increasing library sizes *L*. *L* starts small and grows by a prefixed step. As *L* increases, the points in the manifold become denser and the distance between the nearest neighbours shrinks. This results in reliable value of $\rho$, thus providing a mathematical causal association from *X*(*t*) to *Y*(*t*). A higher $\rho$ indicates a stronger causal association, implying that the state of the predictor variable provides substantial information about the future state of the target variable.

### Analytic approach

The parameter for assessing the causal association between a given employment transition and suicide was $\rho$. $\rho$ is defined as a measure of strength of causality between two time series and can range between -1 to +1. It represents the correlation between observed and predicted values of the causal variable using state space reconstruction of the effect variable. $\rho$ was calculated for the association between changes in employment transition categories and suicide counts was calculated for the association between changes in employment transition categories and suicide counts.

CCM requires a complete set of data between two time series to create shadow manifolds. The overlapping period between the suicide data and employment change data was from January 2009 to December 2018. However, since the December 2018 suicide data was incomplete, it was excluded to avoid bias. Thus, the dataset for analysis spans from January 2009 to November 2018, covering almost 10 years of retrospective data.

CCM involves tuning several hyperparameters, including library size (*L*), embedding dimension (*E*), and time lag ($\tau$). For our analysis, *L* was set between 5 and 50, with increments of 5. *E* was chosen between 3 and 5, and $\tau$ was chosen between -1 and -5. The choice of *L* is governed by the length of the time series, whereas the choices of *E* and $\tau$ were based on past studies that outline the parameter selection^29,30^ and preliminary studies were done using the Empirical Dynamic Modeling vignette in R^31^. A longer $\tau$ could result in oversampling^30^ and a higher *E* could result in over-embedding of the system^29^. In our preliminary study, the parameter $\tau$ was chosen within the range -1 and -10 and it yielded a comparable result compared to -1 to -5. A longer $\tau$ and higher *E* also means smaller *L* because more points are used to reconstruct the shadow manifold which reduces the library length. Hence, trade off was made at -1 to -5 for $\tau$, and 3 to 5 for *E*. The outcome variable $\rho$ was averaged over each *L* with a 95% confidence interval (CI).

All analyses were conducted using the R version 4.4.0^32^, with the package rEDM^33^ for CCM models.

## Data Availability

The monthly variation of employment changes of Australian labour force is publicly available from Australian Bureau of Statistics under GM1 at https://www.abs.gov.au/statistics/labour/employment-and-unemployment/labour-force-australia/jun-2024. Similarly, the monthly death by suicide data of Australian population is publicly available via Australian Institute of Health and Welfare under Deaths due to suicide (Monthly variation) at https://www.aihw.gov.au/suicide-self-harm-monitoring/resources/download-data-tables.
